# A Progressive Nutrient Profiling System to Guide Improvements in Nutrient Density of Foods and Beverages

**DOI:** 10.3389/fnut.2021.774409

**Published:** 2021-12-23

**Authors:** Danielle Greenberg, Adam Drewnowski, Richard Black, Jan A. Weststrate, Marianne O'Shea

**Affiliations:** ^1^PepsiCo, Inc., Research and Development, Purchase, NY, United States; ^2^Center for Public Health Nutrition, University of Washington, Seattle, WA, United States

**Keywords:** nutrient profiling, food quality, food choice, nutritional quality, nutrition policy, energy density, nutrient density

## Abstract

Improving the nutrient density of processed foods is one way to bring the global food supply closer to the WHO Sustainable Development Goals. Nutrient profiling (NP) has emerged as the preferred method of monitoring the progress toward product innovation and reformulation. This paper presents PepsiCo Nutrition Criteria (PNC), a new internal NP model that was designed to guide and monitor improvements in nutrient density and overall nutritional quality of foods and beverages. The new PNC NP model assigns food products into four classes of increasing nutritional value, based on the content of nutrients to limit, along with nutrients and ingredients to encourage. The nutrient standards used for category assignment followed those developed by global dietary authorities. Standards are proposed for calories, sodium, added sugars, saturated, and industrially produced trans fats. Also included are minimum values for food groups to encourage, low-fat dairy, and for country-specific gap nutrients. Internal use of the NP model has spurred product changes that are consistent with WHO goals for industry transparency. An audited review of company products showed that 48% met added sugar, 65% met sodium, and 71% met saturated fat goals. By the end of 2020, in the top 26 regions in which products are sold, 48% of the total sales volume of global beverages had 100 kcal or less from added sugars per 355 ml serving representing 80% of beverage volume and over 90% of food volume sold globally. The PNC NP model is not consumer-facing but is specifically intended for internal use to motivate stepwise and incremental product innovation and reformulation. Transparent and published NP models further WHO goals of engaging industry stakeholders in the (re)formulation of processed foods and beverages consistent with public health goals.

## Introduction

The World Health Organization (WHO) has called on the food industry to reduce the amounts of saturated and trans fats, added sugar, and salt in the global food supply. Noting that “the global burden and threat of non-communicable diseases (NCDs) constitutes a major public health challenge” (1). The WHO pointed to unhealthy diets as the primary NCD risk factor. Based on expert reports, global diets could be improved further through increased consumption of fruits, vegetables, whole grains, legumes, and certain nutrients of need (2–6).

Quantitative methods to assess the nutrient density of foods to meet public health goals are part of a growing scientific field that is known as nutrient profiling or NP (7). NP methods were initially developed to support nutrition and health claims, help regulate marketing and advertising to children, and provide the scientific basis for front of pack labels and logos (8–13). More recently, NP methods have become the basis for product reformulation and the development of more nutrient-dense food products. Developing guidance for industry-driven NP systems to serve as benchmarks for product innovation and reformulation would contribute to the achieving of NCD-related global public health goals (7).

Whereas some NP methods such as Smart Spot (14) or Smart Choices (15) were consumer-centered and educational, other NP systems were developed by the food industry primarily for internal use. For example, Unilever developed a category-specific Nutrition Score System (16, 17) and Nestlé developed the Nestlé Nutritional Profiling System (18–20). While category-specific, both systems were dichotomous, that is individual products either met the pre-set nutrient criteria or they did not. The Choices International program, both dichotomous and category-specific, was unique since it allowed for an introductory phase with a planned imposition of more stringent criteria over 3 years (15). Similarly, the warning label system in Chile (21–23) was introduced with the expectation that more stringent criteria would be progressively imposed with time. That was to allow the industry some time to reformulate food products and to get consumers used to the idea of foods with less saturated fat, added sugar and/or salt (21).

Nutrient profiling schemes with stepwise criteria, still a novel concept, can inform product reformulation goals that are technically feasible while meeting consumer standards of cost, taste and convenience. One such progressive scheme, the PepsiCo Nutrition Criteria (PNC), was specifically developed by PepsiCo for product reformulation and is presented here. Consistent with progressive reformulation goals, stepwise imposition of incremental nutrition criteria was the main driving concept and a unique feature of the PNC model. Applying progressively more stringent nutrition criteria to product reformulation allows for better benchmarking of progress toward nutrition goals. While taking the limits of food technology into account, the PNC addresses nutrients to limit as well as nutrients and food ingredients to encourage.

A secondary goal was to show how the PNC has been used internally to guide product reformulation and product innovation. Some preliminary evaluations of the PepsiCo global product portfolio are included. One of the primary characteristics of the PNC is that there is the incentivization of small changes rather than requiring all or nothing nutrition goals. The PNC guidelines may thus provide a roadmap for others in the food industry to more rapidly make product changes that may benefit the population at large.

Making the PNC more broadly available will permit the tracking and monitoring of companies' progress toward reaching public health nutrition goals. The WHO goals are to limit fat, sugar and salt and to encourage the incorporation in processed foods of nutrients of public health concern, that is vitamins and minerals shown to have shortfalls in the population or segments of the population. Incorporating dietary ingredients of interest (whole grains, fruit, nuts, and seeds) can also make for more nutrient-rich and healthier diets.

The present goal of transparency and disclosure is consistent with the Access to Nutrition Initiative (ATNI) goals to encourage companies to improve the nutrient density of their product portfolios (24). The ATNI Global Index tracks, scores and ranks the world's largest food and beverage manufacturers to promote private sector accountability. Points are awarded to companies for their policies and practice and the level and quality of their public reporting. One question asked by ATNI is whether a company has a published nutrient profiling system that is used to evaluate the nutritional quality of product lines. The PNC is the internal guidance NP system used for innovation and reformulation of products with the goal of producing products with more healthful nutrition profiles.

## Materials and Methods

### Criteria Development

To develop an incremental NP system to help guide the formulation of new foods and beverages or the reformulation of existing ones, a cross-functional team was convened that included internal nutrition scientists and food technologists as well as external independent academic nutrition scientist advisors. The principles that underlay the development of the PNC included external authoritative recommendations from leading global and regional authorities, consideration of the overall diet in the context of individual foods and beverages and published scientific research.

The criteria used a synthesis of nutrition recommendations from global or regional authorities including the recommendations of WHO, Food and Agricultural Organization, US Institute of Medicine, European Food Safety Authority, United States Department of Agriculture Evidence Analysis Library (EAL), Euro diet and country-specific dietary guidelines from the USA, Canada, China, India, Saudi Arabia, Australia, Malaysia, Mexico, Argentina, Chile, Ecuador, Russia, UK, Turkey, France, and South Africa (2, 3, 6, 15, 22–42).

From this synthesis, the generic nutrients to limit were defined (based on a model diet of 2,000 kcal per day), with both saturated fat and added sugars at or below 10% of total energy, sodium at or below 2,000 mg per day, with no partially hydrogenated vegetable oils (PHVOs) and industrially-produced trans fatty acids (iTFA) as low as technically feasible (Table 1).

**Table 1:** Levels for nutrients to limit.

**Nutrients to limit**	Daily reference value^*^	**Published goals (PepsiCo Inc.)**
Calories	2,000	Based on product type and meal Occasion
Saturated fat	≤ 10% kcal	≤ 1.1 g/100 kcal
iTFA	<1% kcal	≤ 0.1 g/100 g, no PHVOs ≤ 0.4 g/100 g
Added sugars	≤ 10% kcal	≤ 10% kcal
Sodium	2,000 mg	≤ 1.0 mg/kcal

*iTFA, industrially produced Trans Fatty Acids; PHVOs, partially hydrogenated vegetable oils*.

Food groups to encourage (FGE) included fruits, vegetables, whole grains, low-fat dairy (dairy product with between 0.5 and 2% milkfat), nuts, seeds, pulses, and legumes. The minimum inclusion in a product was set at $\frac{1}{2}$ of a serving, with the possibility of adding together smaller amounts of multiple food groups to reach $\frac{1}{2}$ of a serving (e.g., $\frac{1}{4}$ of a serving of fruit plus $\frac{1}{4}$ serving of vegetables; Tables 1, 2). The $\frac{1}{2}$ serving was chosen based on previous work on the Healthy Eating Index which noted that to promote dietary variety the number of “different” foods eaten by an individual should be in amounts sufficient to contribute at least one-half of a serving in a food group (19, 43). The amount of whole grains was based on guidance from the Whole Grain Institute and the U.S. Food and Drug Administration (6, 44). For foods, we did not include fruit juices regardless of percentage in these criteria. This was the case no matter what the processing purpose of the fruit juices might have been. For beverages, 100% fruit juice was used as the criteria (which could be fresh or fully reconstituted from concentrate).

**Table 2:** Levels for food groups to encourage.

Fruits	40 g fresh equivalent; 60 ml 100% juice (per serving) is equivalent to $\frac{1}{2}$ serving.
Vegetables	40 g fresh equivalent; 60 ml 100% juice (per serving) is equivalent to $\frac{1}{2}$ serving
Whole grains	8 g (per serving)
Dairy	120 ml milk equivalent; 112 g yogurt; 15 g cheese; 35 g cottage/ricotta cheese (per serving)
Nuts, seeds	15 g (per serving)
Legumes (including pulses)	1/8th cup (25 g) cooked equivalent

### Category Specific Guidelines

**Figure 1:**
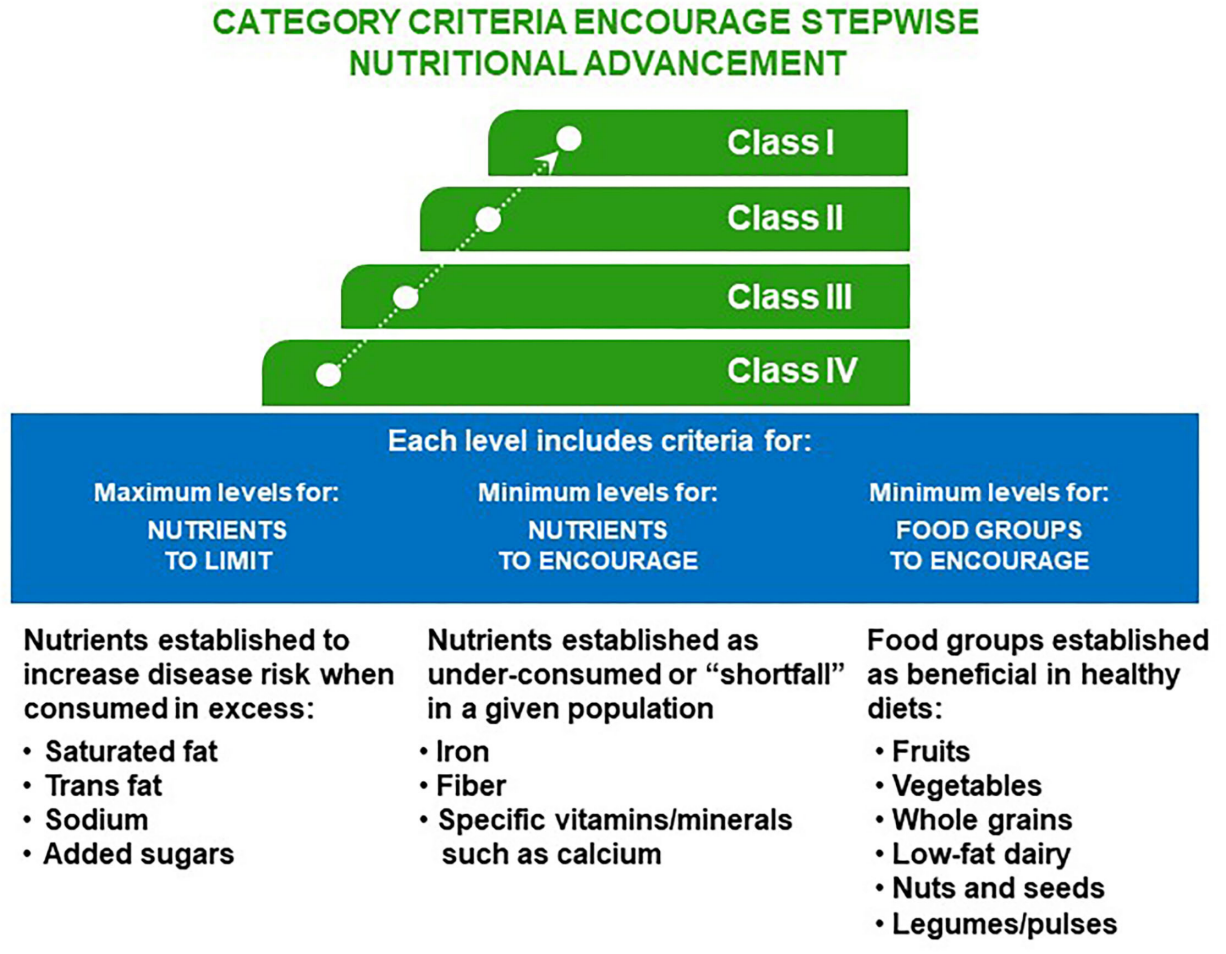
Architecture of PepsiCo Nutrition Criteria (PNC). Nutrient density improves with stepwise progression from Class I to Class IV. Each level is precisely defined with goals of progressively limiting nutrients of concern and augmenting nutrients and food groups to encourage to guide and facilitate product formulation and reformulation in a practical manner.

The PNC were designed to be inclusive of the global portfolio of PepsiCo Inc. foods and beverages and was divided into 20 defined product categories (Table 3). The categorization process was designed to group similar foods and beverages, to identify their role in the diet as well as the typical quantities consumed. Each of the 20 categories has unique stepwise criteria and target levels, or “Classes,” (Class IV, Class III, Class II, Class I; Figure 1). These Class levels (Class IV being the easiest goal to meet to Class I being the hardest to meet) were intended to indicate increasing nutrient density or improvement of the nutritional profile by more stringent limits on added sugars, sodium, or saturated fats. At each level, there are individual requirements for nutrients to limit (NTL), nutrients to encourage (NTE), and FGE (Tables 1, 2, 4, 5 and Supplementary Tables 4A, 5A). The Class levels are designed to encourage stepwise nutritional advancement toward meeting the highest Class level within a category, progressing to the highest level possible within current manufacturing and safety limits. The Class I level nutrient density was intended to be best in class and at minimum meet standards set by global authority guidelines. The purpose of this was to recognize nutrient-dense innovation and inspire progression to keep striving for healthier products even beyond the PNC limits (Table 6). Each additional level from Class IV to Class I was intended to guide product improvement if the highest level (e.g., Class I) was not immediately feasible due to technical challenges and/or consumer acceptance hurdles. Experience has shown that an all-or-nothing approach of meeting or not meeting Class I criteria may, in practicality, lead to fewer rather than more products being reformulated with improved nutrient content. The stepwise gradual approach was intended to lead to a greater number of efforts at a reformulation and set a baseline of nutritional quality for innovation of any new products. It was also possible to achieve progressions on an individual nutrient from Class IV to Class III to Class II to Class I even if the remaining criteria for other nutrients were not feasible. The intent was to immediately improve what was possible with current technology, ultimately improving nutrient density and quality and reducing NTL across a global portfolio with the expectation that further improvements would be made later as novel processing developments become available. For example, sodium reduction can lead to unintended consequences as sodium acts as a humectant and promotes microbial safety (45). Consumer acceptance was another factor to consider in stepwise progression, as significant changes in taste are less acceptable to consumers than small changes over time. These small changes allow for habituation and resultant acceptance. These small changes can also be made without the changes being highlighted for the consumer (46) which again results in greater acceptance. Given that manufacturers are unlikely to make nutritional changes that result in dramatic negative consumer impact the overall result of small changes are that consumer acceptance is maintained and thus overall more changes are made.

**Table 3:** Definitions of product categories based on what we eat in America category codes.

**Category**	**WWEIA 2 digit code**	**WWEIA 4 digit code**	**Long name**
Refreshment beverages	92	2,002–7304	Carbonated soft drinks, non-carbonated beverages, energy drinks, coffee, coffee beverages, tea, tea beverages (not fruit juice or PBB)
Savory or sweet snacks	53,54	5,002–5,204	Potato chips, corn chips, vegetable and fruit chips, popcorn, biscuits, crackers, bread snacks, coated nuts, nut bars
Dips	78	6,422, 8,412	Vegetable based hummus, salsa, tzatziki, guacamole.
Appetizers	54,58		Frozen snacks, other snacks, combination snacks (e.g., crackers and cheese)
Nuts, seeds, nut butters	42	2,804	Whole or shelled tree nuts (>90%), peanuts, coconut/nut/peanut spreads, whole seeds, seed pastes, tahini
Grain foods	50–57	4,002–4,804	Foods containing a pre-set minimum amount of grains and whole grains
Grain beverages	58	-	Beverages containing the minimum amount of grains and whole grains
Dairy beverages	11	1,002–1,404	Milk or products made from or containing a minimum amount of milk
Fruit and vegetable juice	64	7,002–7,008	100% or pure fruit and/or vegetable juices
Fruit and vegetable foods	62,63,72– 75,78	5,002–6,422	Frozen, canned, dried and dehydrated vegetables or fruits, purees, tomato paste. Dried fruits without added salt, fats, added sugars
Combination products	5–7		Snack or beverage product including multiple positive nutrition elements (fruit and vegetables, dairy, grains) for example a dairy based smoothie that contains fruit
Cereals	57	4,802–4,804	Hot, cold, ready-to-eat breakfast cereals including instant oat products and savory cereals
Dairy beverages	11	1,002–1,404	Flavored dairy beverages, mixed dairy and fruit beverages.
Yogurt and dairy desserts	13	1,820–1,822	Yogurt, frozen yogurt ice-cream, pudding, custard, curd, and other desserts.
Side dishes	56	3,202–3,208	Mixture of pasta, rice, cereal grains or vegetables with seasonings/sauce including: rice pilaf, rice and sauce.
Breads, grains, pasta, flours	51, 52, 56	4,002–4,404	Plain pasta, plain rice, pancakes and couscous. Does not include bread snacks
Soup	58, 77	3,802	Soup and ready to eat noodles
Nutrition bars and clusters		5,404	Nutrition bars, clusters, biscuits and nut clusters
Savory foods		5,002–5,008	Nutritious foods that are tangy, salty, or spicy
Meals		3,002–3,602	A combination of >3 food components packaged together providing more calories and nutrition than a mini meal and positioned as an entire meal
Mini-meals		3,702–3,722	A single or combination of >2 food components packaged together providing more calories and nutrition than a snack

*WWEIA, What We Eat In America; PBB, Plant Based Beverages*.

**Table 4:** Definitions for product categories to be used as class guides for product innovation or reformulation.

**Category**	**Definition**	**Examples**	**Exclusions**
Refreshment beverages	Carbonated soft drinks, non-carbonated beverages, energy drinks, coffee, coffee beverages, tea, tea beverages	Carbonated and non-carbonated soft drinks, energy drinks, coffee, and tea	Fruit/veg. juices, dairy/dairy substitutes, plant-based beverages
Savory or sweet snacks	Salty or sweet foods eaten between regular meals	Potato chips, corn chips, vegetable and fruit chips, popcorn, biscuits, crackers, bread snacks, coated nuts, nut bars	Nuts, cereals
Dips	Hummus, salsa, tzatziki, guacamole etc.	Hummus, salsa, tzatziki, guacamole etc.	
Appetizers	Frozen snacks, other snacks that may have food combinations such as crackers and cheese	Frozen snacks, snack food combinations	
Nuts, seeds, nut butters	Products containing at least 90% whole or shelled tree nuts, peanuts, coconut/nut/peanut spreads, whole seeds, seed pastes, tahini	Whole or shelled tree nuts, peanuts, coconut (must contain at least 90% but could be mixed with grains etc.). Nut/peanut spreads, whole seeds, seed pastes, tahini	
Grain foods	Foods containing a proscribed minimum amount of grains and whole grains	Foods with proscribed amount of whole grains	Cereals, breads
Grain beverages	Beverages containing the minimum amount of grains and whole grains	Beverages containing a designated amount of whole grains	Dairy beverages, fruit juices
Dairy beverages	Milk or products made from or containing a minimum amount of milk	Flavored dairy beverages, mixed dairy and fruit beverages	
Fruit and vegetable juices	A beverage product containing a minimum amount of fresh fruit or vegetable equivalent content	100% or pure fruit and/or vegetable juices Can be reconstituted juice diluted w/water/carbonated water. Final juice content is ≥25%	Dairy beverages
Fruit and vegetable foods	Frozen, canned, dried and dehydrated vegetables or fruits, purees, tomato paste. Dried fruits without added salt, fats, added sugars	Frozen, canned, dried dehydrated vegetables or fruits, purees, tomato paste. Dried fruits without added salt, fats, or sugars	Fruit and vegetable juices
Combination products	Snack or beverage product including multiple positive nutrition elements (fruit and vegetables, dairy, grains) for example a dairy based smoothie that contains fruit	Snack or beverage product including multiple positive nutrition elements	Foods solely in one food group
Cereals	Hot, cold, ready-to-eat cereals including instant oat products and savory cereals	Ready-to-eat breakfast cereals, hot cereals, instant grain cereal products, savory cereals	
Yogurt and dairy desserts	Any yogurt, ice cream, custard, curd, etc.	Yogurt, frozen yogurt ice-cream, pudding, custard, curd, and other desserts	20 g whole grain/100 g
Side dishes	Mixture of pasta, rice, cereal grains or vegetables with seasonings/sauce including: rice pilaf, rice and sauce	Mixture of pasta, rice, cereal grains or vegetables with seasonings/sauce including, rice pilaf, rice with sauce, other grains with sauce	Combination products
Breads, grains, pasta, flours	Plain pasta, plain rice, pancakes and couscous. Does not include bread snacks	Plain pasta, plain rice, pancakes and couscous	Bread snacks
Soup	Soup and ready to eat noodles	Soup and ready to eat noodles	Side dishes
Nutrition bars and clusters	Nutrition bars, clusters, biscuits and nut clusters	Nutrition bars, clusters, biscuits, cookies	Breakfast bars
Savory foods	Nutritious foods that are tangy, salty, or spicy	Nutritious foods that are tangy, salty, or spicy	Does not belong to another food category
Meals	A combination of >3 food components packaged together providing more calories and nutrition than a mini meal and positioned as an entire meal	A combination of >3 food components packaged together positioned as an entire meal	Any combination not consisting of at least three different FGEs
Mini-meals	A single or combination of >2 food components packaged together providing more calories and nutrition than a snack	A single or combination of >2 food components packaged together and providing more calories and positive nutrition than snacks	Snacks, individual side dishes

*FGE, food groups to encourage (Additional category definitions that represent less frequently consumed foods or beverages are found in Supplementary Table 4A)*.

**Table 5:** Specific nutrient requirements for all product categories according to PNC nutrient density classification.

**Category**	**Reference quantity**	**Nutrients to limit**	**Criteria for nutrients to limit^a^**	**Stepwise class levels**				**Food groups and/or NTEs (and notes)**
				**Class IV**	**Class III**	**Class II**	**Class I**	
Refreshment beverages	355 ml (12 fl oz)	Calories (kcal)	40					NA Calorie limit meets the definition of low-calorie drinks in most markets
		Saturated fat	NA					
		iTFA (g/100 g)	≤ 0.1					
		Added sugars (g/100 ml)	≤ 3	~7	≤ 5	≤ 3	0	
		Sodium (mg/kcal)	≤ 1.0					
Savory or sweet snacks	**30 g:** Savory snack, nut bars, cookies, nut clusters **40 g:** Grain-based or fruit- based bars **80 g:** Cakes and muffins	Calories (kcal)	≤ 200					≥1 Food group Snacks considered at 10% of daily calories = 200 kcal. Upper limit of saturated fat intake at 10% total dietary energy. For snacks 10% energy from saturated fat = 1.1 g per 100 kcal
		Saturated fat (g/100 kcal)	≤ 0.8	1.7	1.4	1.1	0.8	
		iTFA (g/100 g)	≤ 0.1	NA	NA	<0.1	<0.1	
		Added sugars (kcal)	≤ 10%					
		Sodium (mg/kcal)	≤ 1.0	1.6	1.3	1.0	1.0	
Nuts, seeds, nut butters	**30 g**	Calories (kcal)	≤ 200				NA	Nuts and seeds are considered a plant-based protein source thus is intrinsic in the category Calorie cap of 200 kcal meets recommended levels and limits amount of fat and saturated fat that occur naturally in these foods
		Saturated fat (g/100 kcal)	Prefer nuts/seeds w/ low-saturated fat	≤ 1.7	≤ 1.4	≤ 1.1	≤ 0.8	
		iTFA (g/100 g)	≤ 0.1	NA	NA	≤ 0.1	≤ 0.1	
		Added sugars (kcal)	≤ 10%					
		Sodium (mg/kcal)	≤ 1.0	≤ 1.6	≤ 1.3	≤ 1.0	≤ 1.0	
Dairy and convergence beverages	**240 ml**	Calories	NA	NA	NA	NA	NA	Dairy: FGE should be dairy; NTE should be calcium and 20% of ref amount of calcium Convergence: ≥ 2 FGEs, can be a combination with ≥$\frac{1}{2}$ FGE each Food groups vary by Class level^*^ Calories are limited by the amount of added sugar and fat. Sweetness will come from 100% juices where possible
		Saturated fat (g/100 kcal) Dairy Convergence	≤ 2.5 ≤ 1.5	≤ 3.5	≤ 3.5	≤ 3.5	≤ 1.5	
		iTFA (g/100 g)	≤ 0.1	≤ 0.1	≤ 0.1	≤ 0.1	≤ 0.1	
		Added sugars (g/100 ml)	≤ 3	≤ 7	≤ 5	≤ 3	0	
		Sodium (mg/kcal)	≤ 1.3	≤ 1.6	≤ 1.3	≤ 1.3	≤ 1.3	
Fruit and vegetable juices (includes dairy alternatives, plant- based beverages)	NA	Calories (kcal/100 ml)	<75	<75	<75	<75	<75	>25% of 100% juice per 100 ml or >17 g fresh fruit equivalent per 100 ml and >10% NTE/ serving Food groups vary by Class level^*^
		Saturated fat	NA	NA	NA	NA	NA	
		iTFA	NA	NA	NA	NA	NA	
		Added sugars (g/100 ml)	≤ 7	≤ 7	≤ 5	≤ 3	0	
		Sodium (mg/kcal)	≤ 1.0	≤ 1.0	≤ 1.0	≤ 1.0	≤ 1.0	
Cereals	**30 g:** Breakfast cereals **40 g:** Dry oats **55 g**: High density, flavored, sweetened, or savory dry instant oats, combination cereals (oats with dairy or fruit topping)	Calories (kcal)	≤ 250	≤ 250	≤ 250	≤ 250	≤ 250	16 g whole grain >10% NTE Food groups vary by Class level^*^ Calories based on a breakfast meal of 25% of 2,000 kcal diet
		Saturated fat (g/100 kcal)	≤ 1.1	≤ 1.7	≤ 1.4	≤ 1.1	≤ 0.8	
		iTFA (g/100 g)	≤ 0.1	≤ 0.1	≤ 0.1	≤ 0.1	≤ 0.1	
		Added sugars (kcal)	≤ 10%	≤ 20%	≤ 15%	≤ 10%	≤ 5%	
		Sodium (mg/kcal) Sweet Savory	≤ 1.0 ≤ 1.3	≤ 1.6 ≤ 1.6	≤ 1.3 ≤ 1.3	≤ 1.0 ≤ 1.0	≤ 1.0 ≤ 1.0	
Yogurt and dairy desserts	**225 g:** Yogurt **60 g:** Ice-cream **140 g**: Custards and other desserts	Calories (kcal)	≤ 200	NA	NA	NA	NA	≥2 dairy FGE (224 g yogurt) ≥20% calcium Food groups vary by Class level^*^ Dairy has naturally occurring sodium the limit incorporates this value Calories are constrained by added sugar and saturated fat limits
		Saturated fat (g/100 kcal)	≤ 2.5	≤ 3.5	≤ 3.5	≤ 3.5	≤ 1.5	
		iTFA (g/100 g)	≤ 0.1	≤ 0.1	≤ 0.1	≤ 0.1	≤ 0.1	
		Added sugars (g/100 ml)	≤ 3	≤ 7	≤ 5	≤ 3	≤ 0	
		Sodium (mg/kcal)	≤ 1.3	≤ 1.6	≤ 1.3	≤ 1.3	≤ 1.3	
		Calories (kcal)	≤ 200	≤ 250	≤ 250	≤ 250	≤ 250	
Nutrition bars and clusters	**30 g**: Snacks, cookies, nut barsand nut clusters **40 g:** Grain based and fruit bars 80 g cakes and muffins	Saturated fat (g/100 kcal)	≤ 1.1	≤ 1.7	≤ 1.4	≤ 1.1	≤ 0.8	16 g whole grain or 5 g fiber or >2 NTEs and >2 FGEs Food groups vary by Class level^*^ FGE can be combination from all FGEs not only grains
		iTFA (g/100 g)	≤ 0.1	≤ 0.1	≤ 0.1	≤ 0.1	≤ 0.1	
		Added sugars (kcal)	≤ 10%	≤ 20%	≤ 15%	≤ 10%	≤ 5%	
		Sodium (mg/kcal)	≤ 1.0	≤ 1.6	≤ 1.3	≤ 1.0	≤ 1.0	

a *All values are for finished product*.

* *For details see Supplementary Table 5A*.

*iTFA, industrially produced trans fatty acids; FGE, food group to encourage; NTE, nutrients to encourage (Additional category definitions that represent less frequently consumed foods or beverages are found in Supplementary Table 5A)*.

**Table 6:** Results of category criteria applications.

**Target metrics**	**2016**	**2017**	**2018**	**2019**	**2020**	**2025 target**	**Comments**
≥67% of beverage portfolio volume will have ≤ 100 Calories from added sugars per 12oz. serving by 2025^a^	40%^b^	40%^c^	44%^d^	47%^e^	48%^f^	67%	Global numbers are based on Top 26 Beverage markets, which represent 80% of global beverage volume as of 2020.
≥75% of foods portfolio volume will not exceed 1.3 milligrams of sodium per Calorie by 2025^a^	54%^b^	56%^c^	58%^d^	61%^e^	64%^g^	75%	
≥75% of foods portfolio volume will not exceed 1.1 grams of saturated fat per 100 Calories by 2025^a^	66%^b^	61%^c^	61%^d^	62%^e^	71%^g^	75%	

a *Third-party limited assurance provided (2020 data pending assurance)*.

b *Represents Top 10 markets. Top 10 markets represented 63% of beverages volume and 79% of foods volume as of 2016*.

c *As of 2017, Top 26 Beverage markets represented 80% of our global beverages volume and Top 23 Foods markets represented 90% of our global foods volume*.

d *As of 2018, Top 26 Beverage markets represented 80% of our global beverages volume and Top 23 Foods markets represented 89% of our global foods volume*.

e *As of 2019, Top 26 Beverage markets represented 79% of our global beverages volume and Top 23 Foods markets represented 90% of our global foods volume*.

*Results reflect inclusion of the SodaStream portfolio*.

f *2020 results reflect the inclusion of the SodaStream, Rockstar, and Pioneer Foods portfolios*.

g *2020 results reflect the inclusion of the Pioneer Foods and BFY portfolios*.

### Determination of Effects of PNC on Nutritional Quality of Global Portfolio

Data for sales volume and for the amounts of nutrients to limit and food groups or nutrients to encourage were determined for PepsiCo Inc. products in the top 26 countries or regions and on a global basis. The countries and regions were determined by examining and placing into order by the sales volumes for foods and beverages. Countries with the largest volumes were chosen first. This captured over 80% of total sales volume. Additional regions or countries with the next highest volumes were then added to reach over 97% of total volume sold. The remaining regions or countries contributed such a small percentage of total sales volumes that adding them individually had virtually no effect on the total sales volume and thus they were excluded from this analysis. Data were used both from the years prior to the PNC being introduced and by year since the PNC were introduced. It should be noted that product development meeting PNC criteria was incentivized. All data tracking noted in the results below guided by the PNC criteria were independently audited by The Partnership for a Healthier America (47, 48).

## Results

Individual categories of food and beverage products were assigned criteria for calories, saturated fats, iTFA, added sugars and sodium (NTLs). Some categories had additional specific requirements as noted (Table 3). In addition, positive nutrients were assigned leveraging daily reference values and NTE based on recommendations for dietary intakes issued by numerous authorities including the WHO (7), the US Institute of Medicine (49) and country-specific dietary guidelines (2, 3, 6, 15, 22, 25–42).

Energy had a daily reference value of 2,000 kcal and limits for categories were then based on this value. For example, it is generally accepted in dietary guidelines that an individual snack can contribute up to 10% of daily calories if consumed within a healthy balanced diet and that several planned snacks per day can help improve diet quality (50–56). Thus, based on the 2,000 kcal daily intake the limit for calories for one snack equated to ≤ 200 kcal which was then used as the calorie limit for snack categories. Likewise, we defined calorie limits for all other categories based on recommendations on how that category fits into an overall daily calorie allowance based on authoritative nutritional guidance.

For saturated fat, an upper limit of 10% total dietary energy was used. Therefore, based on a 2,000 kcal diet, 1.1 g per 100 kcal was the saturated fat limit for relevant categories. The limits for trans fats were in line to reduce intake as much as possible and of eliminating PHVOs. Thus, the limit of iTFAs was ≤ 0.1 g/100 g finished product and the resulting limit of PHVOs, was applied to most categories. For products in categories that contain greater amounts of oils, the limit was set so that in the finished product no more than 2% of all oils were iTFAs (1). These goals are being implemented through our stepwise progressions thus not all products meet these goal limits at this time. It should be noted that naturally occurring, and possibly beneficial (57) trans-fats such as those found in some dairy products are not to be excluded from the diet and are therefore not part of the limits.

While there is global consensus on limiting the intake of sugars in the diet, guidelines vary in the specifics of the exact definition of what sugars to limit and whether to include total sugars, added sugars, free sugars, or only non-intrinsic sugars. However, in general, limiting the intakes of added sugars to no more than 10% of total dietary energy is agreed upon and therefore we chose to use the 10% added sugar guidance as our limit (34). We used the definition of added sugars as: all mono- and disaccharides in sugar or sucrose (raw, granulated, powdered, and brown), glucose, dextrose, fructose, lactose (unless naturally present in milk ingredient), maltose, maltodextrin, evaporated cane juice, syrup, corn syrup, corn sweetener, high fructose corn syrup, glucose syrup, malt syrup, brown rice syrup, agave syrup, maple syrup, molasses, invert sugar, icing sugar, honey, fruit sugar syrup and the added sugar component of infused fruits, and other non-specified mono- or di-saccharides. In addition, 100% juice (not from concentrate and from concentrate reconstituted to original levels), as well as sugar alcohols (polyols), are not part of the definition for added sugars that were used. Juice (100%) was classified in this way due to a lack of consensus amongst authorities, insufficient scientific rationale to count as added sugar, and the significant nutrient contribution of juices in the diet. For example, in the US, orange juice is a major contributor of potassium, vitamin C, magnesium, folate and thiamin (58). Sugars from juice concentrate are used to impart sweetness and are not reconstituted to 100% juice and thus were considered added sugars. Authoritative dietary recommendations for sodium differ ranging from 1,600 mg/day to above 2,500 mg/day. A majority of recommendations, however, use 2,000 mg/day as a basis for good health (1, 49). Based on a 2,000 kcal diet, this equates to 1 mg/kcal and thus this limit was implemented in our system. For food groups to encourage (fruits, vegetables, whole grains, low-fat dairy, nuts, seeds, legumes, and pulses), the minimum inclusion in a product was set at $\frac{1}{2}$ of a serving, and as mentioned previously this also had the possibility of adding together a smaller amount of $\frac{1}{4}$ of multiple food groups to reach $\frac{1}{2}$ of a serving. For nutrients to encourage, nutrients were included only if regional authorities had defined them as likely to be at suboptimal intake levels in certain population groups. The inclusion of these positive nutrition goals is especially important in leading to choices of foods that fit optimal dietary patterns. As noted by Tapsell et al. “evidence supporting healthy dietary patterns provides the foundation for the development of dietary guidelines. Further reference to individual foods and nutrients follows from the foundation of healthy dietary patterns” (59). All category and sub-category specific nutrient guidelines are shown in Table 3. Certain categories have specific requirements in addition to the general NTL and inclusion of food groups and NTE. For example, “dairy beverages” have the requirement that the FGE should be dairy, the NTE should be calcium and must be at least 20% of reference amount for calcium. In addition, in the “dairy beverages” category the sodium limits take into consideration the intrinsic sodium in dairy products (Tables 4, 5 and Supplementary Tables 4A, 5A).

The application of the category criteria to the global PepsiCo foods and beverages portfolio have had measurable effects since they were implemented (Table 6). This outcome was supported by the adoption of the criteria globally in a consistent way with commitment from the product development, business and marketing teams to continue to progress and accelerate where feasible. In the Beverage category, there was an expansion of reformulated zero- and lower-calorie beverages. For example, one of the zero sugar products previously sold in only 28 markets in 2015 was expanded to 73 markets at the end of 2017 and over 80 markets in 2020 (48). In terms of stepwise sugar reduction, over 1/3 of products now have formulas with at least 30% less added sugar in 114 countries and regions around the world, replacing the respective full-sugar versions previously in those regions (Table 6). For example, major lemon-lime flavored beverage recipes now contain 30–50% fewer added sugars. Also, a further expansion of zero sugar beverages includes Pepsi Zero Sugar in the US or Pepsi MAX in Western Europe, which was available in 83 markets at the end of 2018, over a 50% increase from 2016. While such substitutions do not necessarily result in sugar reduction, dietary survey analysis using NHANES data demonstrates that consumers of low-calorie sweeteners have overall reduced sugar intake (60). In addition to reformulation efforts, new beverage products are being developed to conform to the added sugars criteria, an effort that is evident in recent portfolio changes in the United States, including a line of flavored sparkling waters with zero sugar and zero added sugar. Zero and reduced sugar options now exist across more product categories, including ready to drink teas, coffees and flavored carbonated and non-carbonated waters. By the end of 2020, in the top 26 countries or regions, 48% of the total global beverage volume had 100 kcal or less from added sugars per 355 ml (12 ounce) serving representing 80% of all global beverage sales (47). This represents an improvement of 8% from the 2015 baseline since the criteria were implemented.

For saturated fats, 71% of PepsiCo's global portfolio does not exceed 1.1 g of saturated fat per 100 kcal in the top 26 markets representing 89% of all food sales (Tables 6, 7) (47, 48, 61–63). Finally, in these top 26 countries or regions, 64% of products do not exceed 1.3 mg/kcal of sodium. This represents an improvement of 10% of products meeting this criterion compared to 2015 (47, 48). This reduction has been accomplished both by reformulation to comply with the category criteria and by introducing new products with less sodium, such as reduced sodium multigrain snacks in global countries or regions (47).

**Table 7:** Examples of products introduced or reformulated to improve nutrient profile.

**Category**	**Product**	**Nature of change**	**Nutrient(s) changed**
Refreshment beverages	Pepsi Zero Sugar	Expanded to 118 countries/regions	Sugar reduction
	7UP, Mirinda and Mt. Dew	Recipies have 30–50% less added sugar in 22 new countries/regions	Sugar reduction
Savory or sweet snacks	Baked Lay's Chips/Snacks	Introduced in 27 new countries/regions	Fat/saturated fat reduction Serving size reduction
	Lays and Kurkure Brand Snacks	Russia and India introduced low sodium and reduced sodium flavors	Sodium reduction Serving size reduction
Grain foods	Introduced Oat Flour in Canada	Whole Grain permitted heart-health claim	
Dairy beverages	Toddynho Levinho in Latin America	Traditional beverage supplies added nutrients low added sugar (7 g/200 ml serving)	Added ≥15% DV calcium Added ≥15% Vitamin D Added ≥15% folic acid Added ≥15% Vitamin A Added ≥15% Vitamin C
Fruit and vegetable juices	Naked Half Naked	100% Juice Mixture of 100% juice and coconut water	Zero added sugar, total sugar (from juice) 27 g/15.2 fl oz serving
Fruit and vegetable foods	Chickpea Veggie Crisps	Pulse based snack product	0 g fat/saturated fat 2 g total sugar 0 g added sugar
Cereals	3 Minutos in Mexico	Fortified 100% Whole Grain cereal with needed nutrients	Added 10% DV calcium Added 11% DV Vitamin A (Mexico gap nutrient)

It is more difficult to assess progress toward meeting positive nutrition criteria, as this covers a wider range of products and sales data do not necessarily track these improvements. However, no food company relies on a single program (such as PNC) to drive portfolio change. Sales growth of oat, nut, seed, or legume based products (specific FGE) are being driven primarily by consumer demand and thus the portfolio has shown a marked expansion of a wide range these products (64).

## Discussion

To meet the recommendations of WHO and ATNI for industry transparency we have described here the NP system that PepsiCo has implemented (1, 7, 24). The creation and implementation of the PNC allow translation of global dietary recommendations into criteria for specific product development goals within a context of manufacturing techniques that are currently available. The system incorporates not only NTL but also promotes the incorporation of both food groups and NTE. The primary goal of this system is to guide product development to be more in line with global nutrition recommendations and thus positioning products to better meet individual requirements. The secondary goal of the system is to drive these changes as rapidly as possible. It is the stepwise nature of the PNC that makes attaining this goal more likely. This system could thus be fairly easily implemented by many companies in the food industry. Product developers and commercialization teams use the criteria to guide both renovations of existing brands and for the development of new products, with the ultimate goal of transforming the global portfolio to be more in line with publicly stated nutrition goals.

Using the PNC, conceptually and practically, could drive industry consistency of new and reformulated products. Implementation of this system has produced measurable significant progress toward improved nutrition goals to date. Progress is not only at an individual product level but also across the portfolio. To ensure significant transformation, the company set challenging public goals for the reduction of nutrients to limit by 2025 across its global food and beverage portfolio (48). For example, since the implementation of the PNC notable progress has been made on the global goal to reduce added sugars across the global beverage portfolio. For added sugars, the 2025 goal is that 67% of the total sales volume of beverages will not exceed 100 kcal per 355 ml (12 ounces), and we continue to make progress toward this goal. For saturated fat, the 2025 goal is that 75% of total foods volume will not exceed 1.1 g of saturated fat per calorie, and currently 71% of the volume of the food meet this goal. Finally, for sodium, the 2025 goal is that 75% of total foods volume will not exceed 1.3 mg of sodium per calorie, and currently 64% of foods meet this goal.

Since the PNC is a science-based system that follows governmental nutrition guidelines and translates these guidelines into meaningful criteria for a wide range of food and beverage products, utilizing it for several purposes is possible. The emphasis on positive nutrition is extremely important and ultimately the goal of all NP systems is to improve the quality of the diet of individuals and as such requires more than just limiting certain nutrients.

There are some limitations of the current PNC that will need expansion in the future. For example, the PNC is not currently designed to meet the needs of special populations such as women of childbearing age, adolescents, or older adults. These populations have special nutrition needs and it will be important to address this in future versions of the PNC especially for products that are targeted to these population segments. While recognizing this is important, it is expected that the current system will be beneficial for all subpopulations. In addition, any such system will need to be updated regularly to respond to changes in dietary guidance. The PNC is not intended to be consumer-facing and is not designed to educate consumers on the nutritional quality of products, unlike other NP schemes. Like all NP schemes, the potential intended impact on public health is challenging to demonstrate due to numerous factors inconsistency in product composition across global markets, and independent factors impacting public health. Therefore, the public health impact of the PNC is not an outcome that was feasible to include in this paper.

One of the aims of this publication is to make transparent not only the nutrition criteria but also how they are being utilized to promote product reformulation and innovation. As noted previously the effects of implementing the PNC on NTL are being independently evaluated by the PHA, an organization that works with the private sector to help improve health and reduce childhood obesity.

In the end, the approach is validated such that the portfolio it has been applied to has improved in its nutritional quality to enable choices to meet changing consumer needs. Yet there is still significant progress to be made in the transformation of the product portfolio, not only to reach stated 2025 company goals but moreover to provide individuals with more nutrient-dense choices to support improved dietary patterns. By having a transparent process that promotes reducing added sugars, saturated fat and salt, while encouraging key food groups and leads to the development of a broader portfolio of product choices we hope to enable a broader focus on positive nutrition.

The initial success of the PNC system suggests the possibility of a potential model for the improvement of the processed food supply. For example, small companies that are unable to develop their systems could easily adopt the PNC system as it applies to most food categories sold by the food industry. More broadly as recently noted by Drewnowski et al. the importance of healthy food choices and dietary patterns is a major emphasis of dietary recommendations (65). These emphasize food groups and dietary ingredients rather than specific nutrients and “nutrient profiling models provide a quantitative tool to guide these policies and evaluate their effectiveness” (65).

Drewnowski et al. also highlight the need for profiling systems that go beyond a focus on specific NTL (fat, saturated fats, sugars, and salt), extending to the total nutrient density of the food or beverage (65). The PNC is such a system, with most categories requiring food groups to encourage be included in the product. Importantly the PNC's incremental stepwise goals facilitate improvements on a practical time scale that systems that are “all or nothing” are unlikely to match. We hope that others will adopt similar stepwise systems that will result in timely offerings to consumers that lead to the greatest possible health improvements.

While the practice of developing and implementing these criteria into the innovation process of a large CPG company is challenging, it is also very worthwhile in terms of the cumulative value on improving the nutritional profile of the product portfolio for consumers globally. Continued efforts across the industry of food and beverage manufacturers to reformulate and innovate new products with improved nutrient profiles will be necessary to help support consumers with choices to improve their diet and health.

## Conclusions

We have developed a novel NP system that considers nutrients to limit, nutrients to encourage, food groups to encourage and sets category-specific incremental step-wise goals to facilitate rapid changes in food and beverage products. Making public this NP system meets WHO and ATNI stated recommendations for industry transparency.

The incremental nature of the current system encourages reformulation and innovation of products within practical manufacturing considerations. The system is a possible industry road map for guiding product development and reformulation to better meet well-agreed-upon population dietary goals with a focus on positive nutrition.

## Data Availability Statement

Publicly available datasets were analyzed in this study. This data can be found at: https://www.pepsico.com/sustainability-report/downloads.

## Author Contributions

All authors contributed to the design of this research, analysis of the data, writing, reading, and approval of the final manuscript.

## Funding

This study was fully funded by PepsiCo Inc. The funders had no role in study design, data collection and analysis, or preparation of the manuscript. The funders approved the submitted manuscript prior to submission.

## Conflict of Interest

DG, RB, JW, and MO'S were full-time employees of PepsiCo Inc. at the time this research was performed. AD was the originator of the Nutrient Rich Food Index, an early NP model, has received grants, contracts, and honoraria from entities both public and private with an interest in nutrient profiling and (re) formulation of foods, and served as a consultant to PepsiCo Inc. for this project.

## Publisher's Note

All claims expressed in this article are solely those of the authors and do not necessarily represent those of their affiliated organizations, or those of the publisher, the editors and the reviewers. Any product that may be evaluated in this article, or claim that may be made by its manufacturer, is not guaranteed or endorsed by the publisher.
