# Supplementary material for: A Progressive Nutrient Profiling System to Guide Improvements in Nutrient Density of Foods and Beverages
Source: Front Nutr. 2021 Dec 23;8:774409. doi: 10.3389/fnut.2021.774409 (PMC8733001; doi:10.3389/fnut.2021.774409)
Supplement: Supplementary file 1 [file Table_1.docx]

**Table 4a –**

**Definitions for product categories to be used as class guides for product innovation or reformulation (supplemental material)**

|  | **Reference Quantity** | **Nutrients To Limit** | | **Criteria For Nutrients  To Limit^1^** | **Specific Class Levels *** | | | | | | | **Food Groups and/or Nutrients to Encourage (& Notes)** |
| --- | --- | --- | --- | --- | --- | --- | --- | --- | --- | --- | --- | --- |
| **Category** |  |  |  |  | **Class IV** | | **Class III** | | **Class  II** | | **Class  I** |  |
| **Dips** | Must adhere to regional regulations | Calories (kcal) | | <200 |  | |  | |  | |  | ≤1 food group  Calories will not exceed 200 kcal (10% of a 2,000 kcal daily intake) |
|  |  | Saturated fat (g/100 kcal) | | ≤0.8 | 1.7 | | 1.4 | | 1.1 | | 0.8 |  |
|  |  | iTFA (g/100 g) | | ≤0.1 | NA | | NA | | ≤0.1 | | ≤0.1 |  |
|  |  | Added sugars (kcal) | | <10% | NA | | NA | | NA | | NA |  |
|  |  | Sodium (mg/kcal) | | ≤1.0 | 1.6 | | 1.3 | | 1.0 | | 1.0 |  |
| **Appetizers** | **85 g** | Calories (kcal) | | ≤200 |  | |  | |  | |  | Calories will not exceed 200 kcal (10% of a 2,000 kcal daily intake) |
|  |  | Saturated fat (g/100 kcal) | | ≤0.8 | 1.7 | | 1.4 | | 1.1 | | 0.8 |  |
|  |  | iTFA (g/100 g) | | ≤0.1 | NA | | NA | | <0.1 | | <0.1 |  |
|  |  | Added sugars (kcal) | | ≤10% | NA | | NA | | NA | | NA |  |
|  |  | Sodium (mg/kcal) | | ≤1.0 | 1.6 | | 1.3 | | 1.0 | | 1.0 |  |
| **Grain Beverages** | **240 ml** | Calories (kcal/30 g) | | Not limited as based on ingredients |  | |  | |  | | NA | 7 g whole grain/100 ml and/or combination of grains and fortification to meet 10% reference amount of fiber and folate  Varies by medal level^*^ |
|  |  | Saturated fat (g/100 kcal) | | ≤0.8 | ≤1.7 | | ≤1.4 | | ≤1.1 | | ≤0.8 |  |
|  |  | iTFA (g/100 g) | | ≤0.1 | ≤0.1 | | ≤0.1 | | ≤0.1 | | ≤0.1 |  |
|  |  | Added sugars (g/100 ml) | | 0 | ≤7 | | ≤5 | | ≤3 | | 0 |  |
|  |  | Sodium (mg/kcal) | | ≤1.0 | ≤1.6 | | ≤1.3 | | ≤1.0 | | ≤1.0 |  |
| **Grain Foods** | **100 g** | Calories | | NA | ≤250 | | ≤250 | | ≤250 | | ≤250 | 20 g whole grain  >10% NTE  Varies by medal level^*^ |
|  |  | Saturated fat (g/100 kcal) | | ≤1.1 | ≤1.7 | | ≤1.4 | | ≤1.1 | | ≤0.8 |  |
|  |  | iTFA (g/100 g) | | ≤0.1 | ≤0.1 | | ≤0.1 | | ≤0.1 | | ≤0.1 |  |
|  |  | Added sugars (kcal) | | <10% | ≤20% | | ≤15% | | ≤10% | | ≤5% |  |
|  |  | Sodium (mg/kcal) | | ≤1.3 | ≤1.6 | | ≤1.3 | | ≤1.0 | | ≤1.0 |  |
| **Fruit and Vegetable Foods** | Regionally appropriate | Calories (kcal/100 g) | | <75 | ≤75 | | ≤75 | | ≤75 | | ≤75 | >34 g fresh fruit or vegetable equivalent per 100 g and  ≥1 NTE  Varies by medal level^*^  Positive nutrition based on full serving of fruit or vegetable |
|  |  | Saturated fat | | NA | NA | | NA | | NA | | NA |  |
|  |  | iTFA | | NA | NA | | NA | | NA | | NA |  |
|  |  | Added sugars (g/100 g) | | ≤3 | ≤7 | | ≤5 | | ≤3 | | 0 |  |
|  |  | Sodium (mg/kcal) | | ≤1.0 | ≤1.0 | | ≤1.0 | | ≤1.0 | | ≤1.0 |  |
| **Combination Product** | Will vary depending on product composition | Calories | | No specific limit |  | |  | |  | |  | Two FGEs or a combination of one FGE per serving  Varies by medal level*  Positive nutrition related to 2 servings of FGE. Limits are per unit volume for beverages and unit mass for foods (for PNC and stepwise limits for this category) |
|  |  | Saturated fat (g/100 kcal) | | ≤1.5 | ≤2.5 | | ≤2.5 | | ≤1.5 | | ≤1.5 |  |
|  |  | iTFA (g/100 g) | | ≤0.1 | ≤0.1 | | ≤0.1 | | ≤0.1 | | ≤0.1 |  |
|  |  | Added sugars (g/100 ml) | | ≤3 | ≤7 | | ≤5 | | ≤3 | | 0 |  |
|  |  | Sodium (mg/kcal) | | ≤1.3 | ≤1.6 | | ≤1.3 | | ≤1.3 | | ≤1.3 |  |
| **Side Dishes** | Will vary depending on ingredients e.g. vegetable ingredients permit larger serving size | Calories (kcal) | | NA | NA | | NA | | NA | | NA | Two FGEs per serving  Varies by medal level*  Positive nutrition based on 2 servings of any FGE |
|  |  | Saturated fat (g/100 kcal) | | ≤1.1 | ≤1.7 | | ≤1.4 | | ≤1.1 | | ≤0.8 |  |
|  |  | iTFA (g/100 g) | | ≤0.1 | ≤0.1 | | ≤0.1 | | ≤0.1 | | ≤0.1 |  |
|  |  | Added sugars (g/100 g-ml) | | ≤3 | ≤7 | | ≤5 | | ≤3 | | ≤0 |  |
|  |  | Sodium (mg/serving) | | ≤400 | ≤500 | | ≤500 | | ≤400 | | ≤400 |  |
| **Breads, Grains, Pasta, Flours** | **40 g** | Calories (kcal) | | NA | NA | | NA | | NA | | NA | 40 g whole grain/100  Varies by medal level*  8 g whole grain per serving |
|  |  | Saturated fat (g/100 kcal) | | ≤0.8 | ≤1.7 | | ≤1.4 | | ≤1.1 | | ≤0.8 |  |
|  |  | iTFA (g/100 g) | | ≤0.1 | ≤0.1 | | ≤0.1 | | ≤0.1 | | ≤0.1 |  |
|  |  | Added sugars (kcal) | | ≤10% | ≤20% | | ≤15% | | ≤10% | | ≤5% |  |
|  |  | Sodium (mg/kcal) | | ??? | ≤1.6 | | ≤1.3 | | ≤1.0 | | ≤1.0 |  |
| **Soup** | Will vary depending on ingredients e.g. vegetable ingredients permit larger serving size | Calories (kcal) | NA | | NA | NA | | NA | | NA | | 2 FGEs per serving  Varies by medal level*  Positive nutrition based on 2 servings of any FGE |
|  |  | Saturated fat (g/100 kcal) | ≤1.1 | | ≤1.7 | ≤1.4 | | ≤1.1 | | ≤0.8 | |  |
|  |  | iTFA (g/100 ml) | ≤0.1 | | ≤0.1 | ≤0.1 | | ≤0.1 | | ≤0.1 | |  |
|  |  | Added sugars (g/100 ml) | <3 | | ≤7 | ≤5 | | ≤3 | | 0 | |  |
|  |  | Sodium (mg/serving) | ≤400 | | ≤500 | ≤500 | | ≤400 | | ≤400 | |  |
| **Savory Foods** | Will vary depending on main food group | Calories (kcal) | NA | | NA | NA | | NA | | NA | | 2 FGEs per serving  Positive nutrition based on 2 servings of any FGE |
|  |  | Saturated fat (g/100 kcal) | ≤1.1 | | ≤1.7 | ≤1.4 | | ≤1.1 | | ≤0.8 | |  |
|  |  | iTFA (g/100 g) | ≤0.1 | | ≤0.1 | ≤0.1 | | ≤0.1 | | ≤0.1 | |  |
|  |  | Added sugars (g/100 g) | ≤3 | | ≤7 | ≤5 | | ≤3 | | 0 | |  |
|  |  | Sodium (mg/serving) | ≤400 | | ≤500 | ≤500 | | ≤400 | | ≤400 | |  |
| **Meals**  **(note as this is a new category no stepwise goals are in place)** | Will vary depending on reference quantity of components | Calories (kcal/ref quantity) | <500 | |  |  | |  | |  | | Will contain ≥3 FGEs per reference quantity.  The 3 FGEs should have ≥2 different FGEs.  Each should contribute at least ½ of an FGE reference quantity.  Reference quantity derived from US FDA Reference Amounts Commonly Consumed |
|  |  | Saturated fat (g/100 kcal) | ≤1.5 | |  |  | |  | |  | |  |
|  |  | iTFA (g/100 g) | <0.1 | |  |  | |  | |  | |  |
|  |  | Added sugars (kcal) | ≤10% | |  |  | |  | |  | |  |
|  |  | Sodium (mg/kcal)  Sweet  Savory | ≤1.0 ≤1.3 | |  |  | |  | |  | |  |
| **Mini-Meals**  **(note as this is a new category no stepwise goals are in place)** | Will vary depending on reference quantity of components | Calories (kcal/ref quantity) | <350 | | NA | NA | | NA | | NA | | Will contain ≥2 FGEs per reference quantity at a meaningful level.  The 2 FGEs should have different FGEs.  Each should contribute at least ½ of an FGE reference quantity.  Reference quantity derived from US FDA Reference Amounts Commonly Consumed |
|  |  | Saturated fat (g/100 kcal) | ≤1.1 | |  |  | |  | |  | |  |
|  |  | iTFA (g/100 g) | ≤0.1 | |  |  | |  | |  | |  |
|  |  | Added sugars (kcal) | ≤10% | |  |  | |  | |  | |  |
|  |  | Sodium (mg/kcal)  Sweet  Savory | ≤1.0 ≤1.3 | |  |  | |  | |  | |  |

1. All values are for finished product

* For details see Table 5a

Abbreviations: FGE= food groups to encourage; NTE=nutrients to encourage; iTFA= industrially produced trans fatty acids; PNC=PepsiCo Nutrition Criteria; US FDA= United States Food and Drug Administration
