# Supplementary material for: A Progressive Nutrient Profiling System to Guide Improvements in Nutrient Density of Foods and Beverages
Source: Front Nutr. 2021 Dec 23;8:774409. doi: 10.3389/fnut.2021.774409 (PMC8733001; doi:10.3389/fnut.2021.774409)
Supplement: Supplementary file 2 [file Table_2.docx]

**Table 5a Supplemental Material**

**Specific nutrient requirements for all product categories according to PNC nutrient density classification**

**and notes for product categories**

| **Category** | **Class IV** | **Class III** | **Class II** | **Class I** |
| --- | --- | --- | --- | --- |
| Dips | NA | NA | NA | NA |
| Appetizers | NA | NA | NA | NA |
| Grain Beverages | 3.5 g whole grain/100 ml | 3.5 g whole grain/100 ml | 7 g whole grain/100 ml | 7 g whole grain/100 ml |
| Grain Foods | 20 g whole grain/100 g | 20 g whole grain/100 g | 40 g whole grain/100 g | 40 g whole grain/100 g |
| Fruit and  Vegetable Foods | 17 g fresh fruit or vegetable equivalent per 100 g and ≥10% NTE/serving | 17 g fresh fruit or vegetable equivalent per 100 g and ≥10% NTE/serving | 34 g fresh fruit or vegetable equivalent per 100 g AND ≥ 20% NTE/serving | 34 g fresh fruit or vegetable equivalent per 100 g AND ≥ 20% NTE/serving |
|  | (Positive nutrition based on ½ serving of fruit or vegetable) | (Positive nutrition based on ½ serving  of fruit or vegetable) | (Positive nutrition based on full serving of fruit or vegetable) | (Positive nutrition based on full serving  of fruit or vegetable) |
| Combination  Product | 1 FGE or a combination of one FGE per serving | 2 FGEs or a combination of one FGE per serving | 2 FGEs or a combination of FGEs to equate to two FGEs per serving | 3 FGEs or a combination of one FGE  per serving |
|  | (Positive nutrition based on 1 serving of any FGE. It is possible to have combinations of FGEs to achieve  the target.) | (Positive nutrition based on 2 servings of any FGE. It is possible to have combinations of FGEs to achieve  the target.) | (Positive nutrition based on 2 servings of any FGE. It is possible to have combinations of FGEs to achieve  the target.) | (Positive nutrition based on 3 servings  of any FGE. It is possible to have combinations of FGEs to achieve the target. No expectation of specific FGEs.) |
| Side Dishes | 1 FGE per serving | 1 FGE per serving | 2 FGEs per serving | 2 FGEs per serving |
|  | (Positive nutrition based on 1 serving of any FGE) | (Positive nutrition based on 1 serving of any FGE) | (Positive nutrition based on 2 servings of any FGE) | (Positive nutrition based on 2 servings  of any FGE) |
| Breads, Grains,  Pasta, Flours | Whole grain: 20 g/100 g  At least 8 g per serving | Whole grain: 20 g/100 g  At least 8 g per serving | Whole grain: 40 g/100 g  At least 16 g per serving | Whole grain: 40 g/100 g  At least 16 g per serving |
| Soup | 1 FGE per serving | 1 FGE per serving | 2 FGEs per serving | 2 FGEs per serving |
|  | (Positive nutrition based on 1 serving of any FGE) | (Positive nutrition based on 1 serving of any FGE) | (Positive nutrition based on 2 servings of any FGE) | (Positive nutrition based on 2 servings  of any FGE) |
| Savory Foods | NA | NA | NA | NA |
| Meals  Note: as this is a new category no stepwise goals are needed | |  |  |  |
| Mini-Meals  Note: as this is a new category no stepwise goals are needed | |  |  |  |

Abbreviations: NTE= Nutrients to encourage; FGE= food groups to encourage;
